# Supplementary material for: The origin of the boundary strengthening in polycrystal-inspired architected materials
Source: Nat Commun. 2021 Jul 29;12:4600. doi: 10.1038/s41467-021-24886-z (PMC8322276; doi:10.1038/s41467-021-24886-z)
Supplement: Supplementary file 1 — Supplementary Information [file 41467_2021_24886_MOESM1_ESM.pdf]

Supplementary Information

The origin of the boundary strengthening in polycrystal-inspired architected materials

Chen Liu, Jedsada Lertthanasarn & Minh-Son Pham*

Supplementary Table 1. Calculated and measured relative densities of meta-crystals and their differences

Supplementary Figure 1. Illustration of lattice orientations and construction of a singly oriented meta-crystal.

Supplementary Figure 2. Mechanical behaviour of FCC35.26.

Supplementary Figure 3. Lattice model and set-up for FEA simulation.

Supplementary Figure 4. Illustration of internal boundary between two meta-grains.

Supplementary Figure 5. Reproducibility of stress-strain response of architected meta-crystals.

Supplementary Table

Supplementary Table 1. Calculated and measured relative densities of meta-crystals and their differences

No. of meta-grains	1	2	4	8	16	32	64
Calculated	0.288	0.295	0.302	0.303	0.321	0.334	0.328
Measured	0.302	0.301	0.324	0.323	0.346	0.370	0.364
Difference (%)	4.9	2.0	7.3	6.6	7.8	10.8	11.0

Supplementary Figures

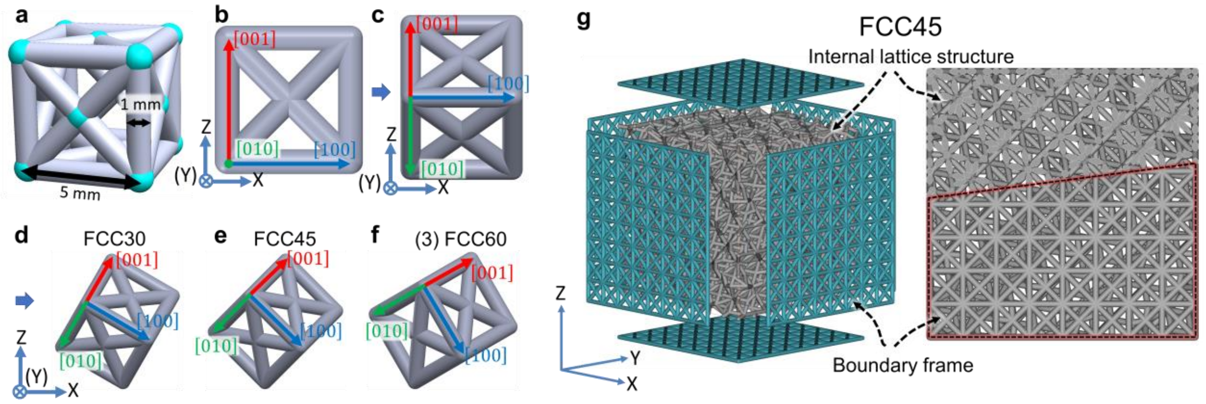

Supplementary Figure 1. Illustration of lattice orientations and construction of a singly oriented meta-crystal. a The dimension of FCC unit cell. **b** Initial position of unit cell with reference to the global orthogonal axes. **c** First rotation by 45° about the X-axis. **d-f** Second rotation by θ about the Y-axis, where θ = 30°, 45° and 60°. **g** Global lattice structure of FCC45 with demonstration of internal lattice structure and boundary frame structures.

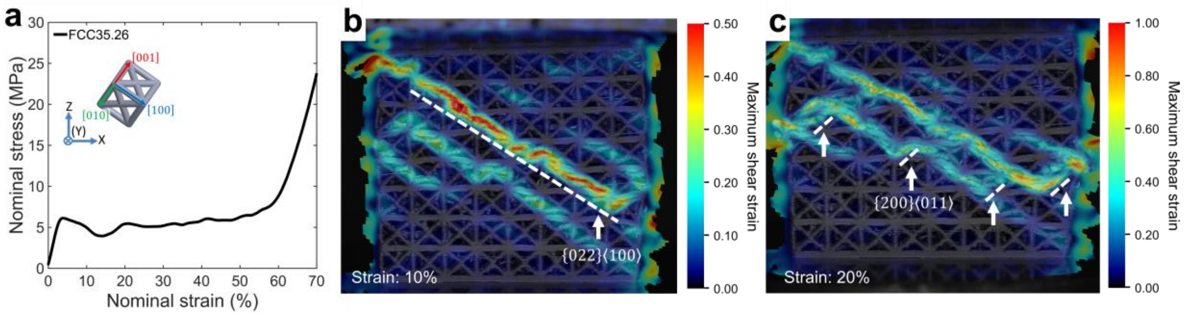

Supplementary Figure 2. Mechanical behaviour of FCC35.26. a Stress-strain response. **b** and **c** Spatial deformation behaviours revealed via DIC analyses at 10% and 20% strains respectively.

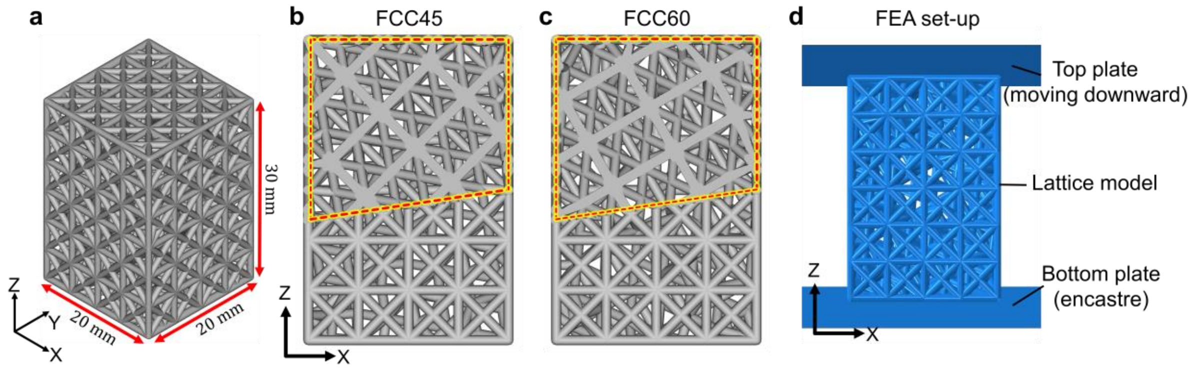

Supplementary Figure 3. Lattice model and set-up for FEA simulation. a Global dimensions of FE lattice model. **b** FCC45, where partial surface frame was removed to show the internal lattice orientation. **c** FCC60. **d** Boundary conditions for FEA.

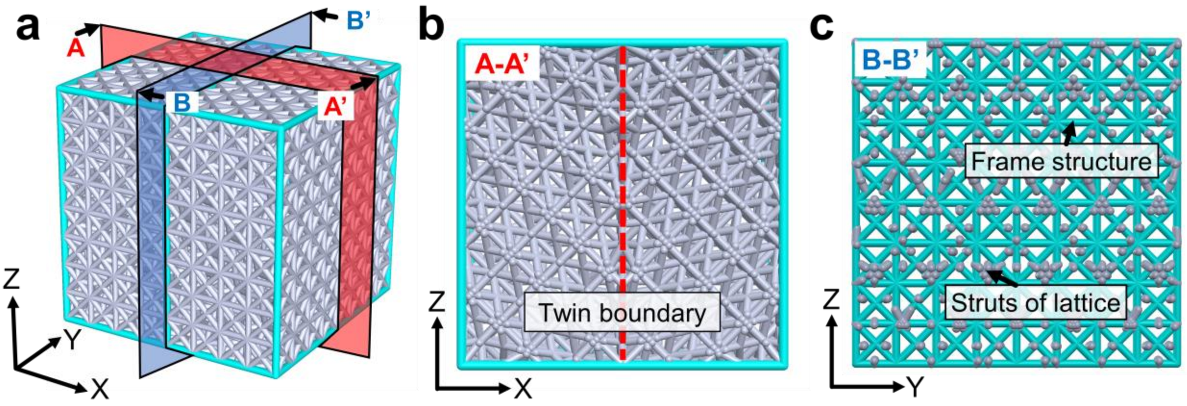

Supplementary Figure 4. Illustration of internal boundary between two meta-grains. a Meta-crystal containing 2 meta-grains of the FCC45. **b** A-A' cross-sectional view highlighting the location of a twin boundary shown in (a). **c** B-B' section of (a) showing the 2D frame (highlighted by light green colour) which acts as the boundary (noted: lattice struts connected to the boundary were shown in grey).

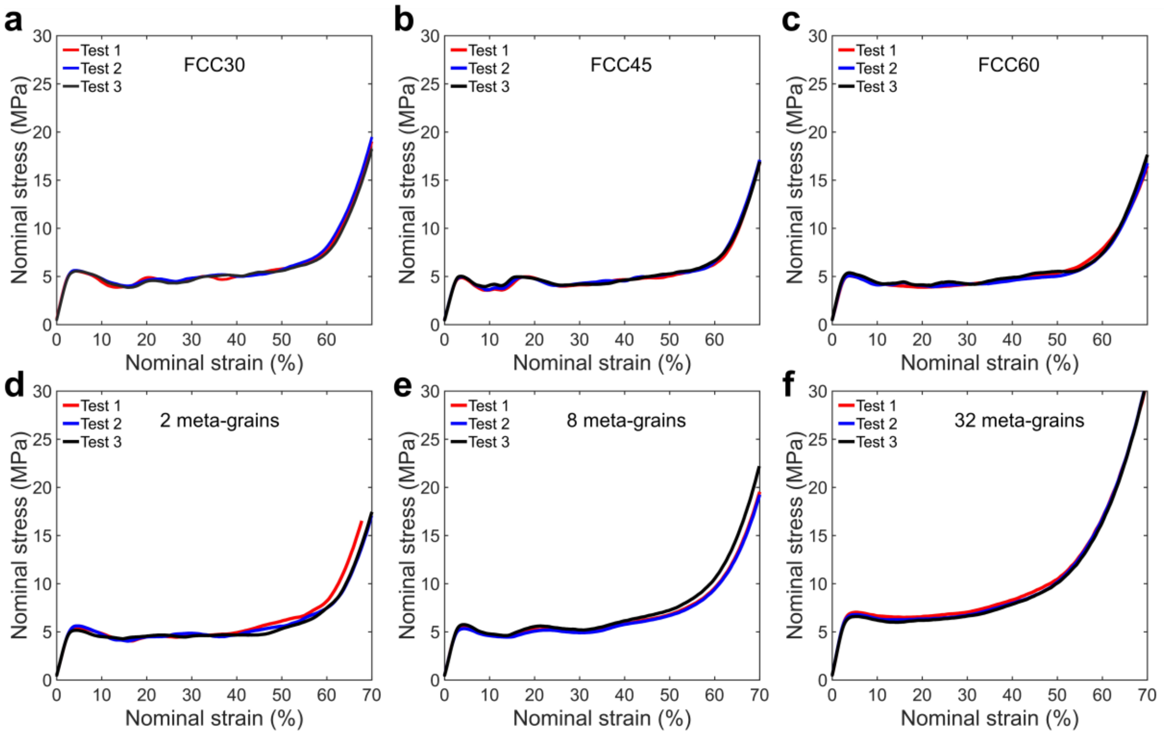

Supplementary Figure 5. Reproducibility of stress-strain response of architected meta-crystals. a FCC30. **b** FCC45 (i.e. single grain meta-crystal). **c** FCC60. **d** 2 meta-grain. **e** 8 meta-grains. **f** 32 meta-grains.
