# Supplementary material for: The origin of the boundary strengthening in polycrystal-inspired architected materials
Source: Nat Commun. 2021 Jul 29;12:4600. doi: 10.1038/s41467-021-24886-z (PMC8322276; doi:10.1038/s41467-021-24886-z)
Supplement: Supplementary file 3 — Description of Additional Supplementary Files [file 41467_2021_24886_MOESM3_ESM.pdf]

Description of Additional Supplementary Files

File Name: Supplementary Movie 1
Description: Deformation behaviour of single meta-crystal with strain distribution identified by colours.

File Name: Supplementary Movie 2
Description: Deformation behaviour of polycrystal-like meta-crystal containing 64 meta-grains with strain distribution identified by colours.
